# Supplementary material for: The Catalytic Role of Superparamagnetic Iron Oxide Nanoparticles as a Support Material for TiO2 and ZnO on Chlorpyrifos Photodegradation in an Aqueous Solution
Source: Nanomaterials (Basel). 2024 Feb 1;14(3):299. doi: 10.3390/nano14030299 (PMC10856829; doi:10.3390/nano14030299)
Supplement: Supplementary file 1 [file nanomaterials-14-00299-s001.zip › nanomaterials-2815865-supplementary.pdf]

Supplementary information

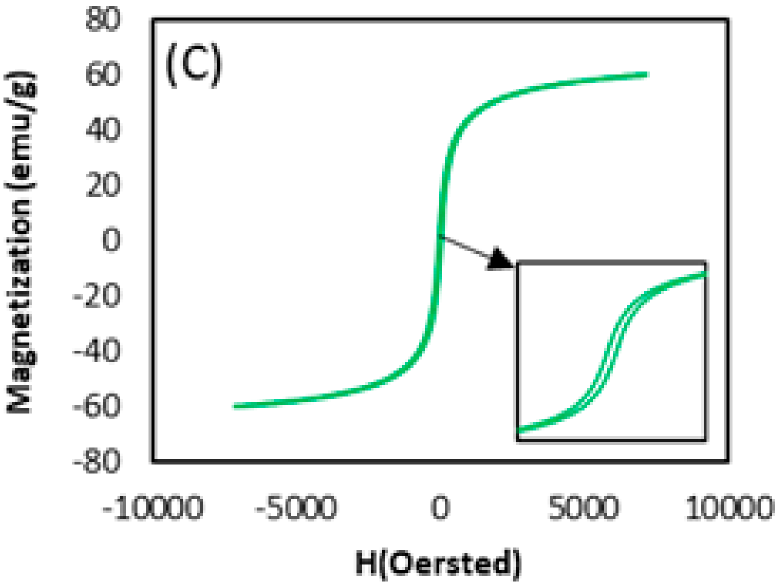

Figure S1. Hysteresis loop of Fe3O4 NP and inset at 0 Oe obtained by VSD.

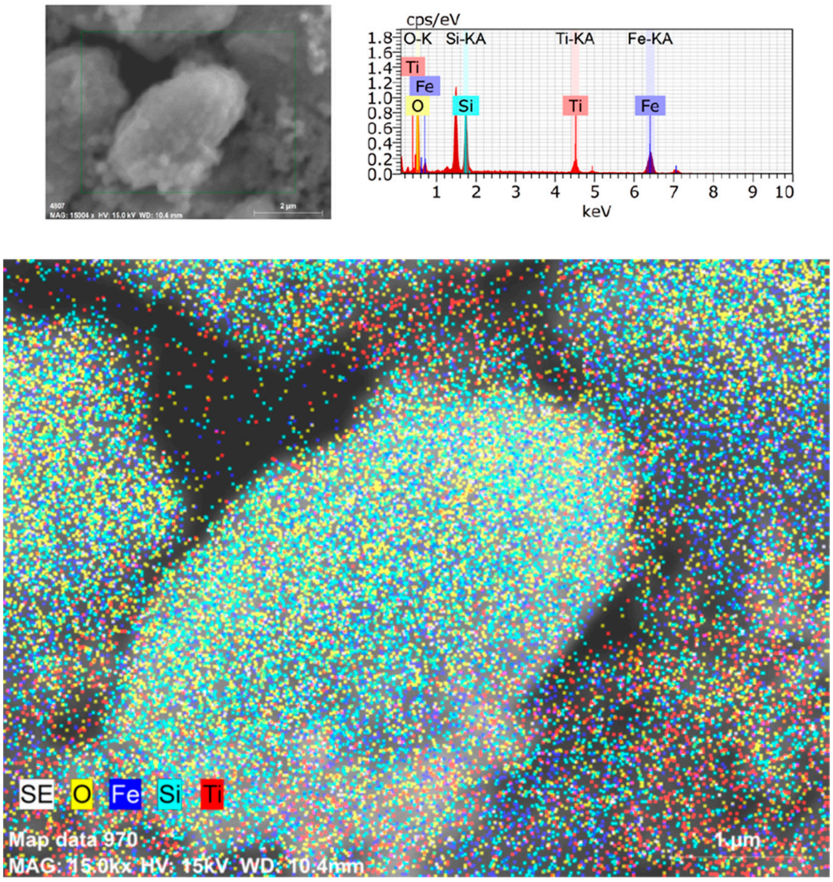

Figure S2. Energy-dispersive X-ray spectroscopy of $Fe_3O_4@SiO_2@TiO_2$

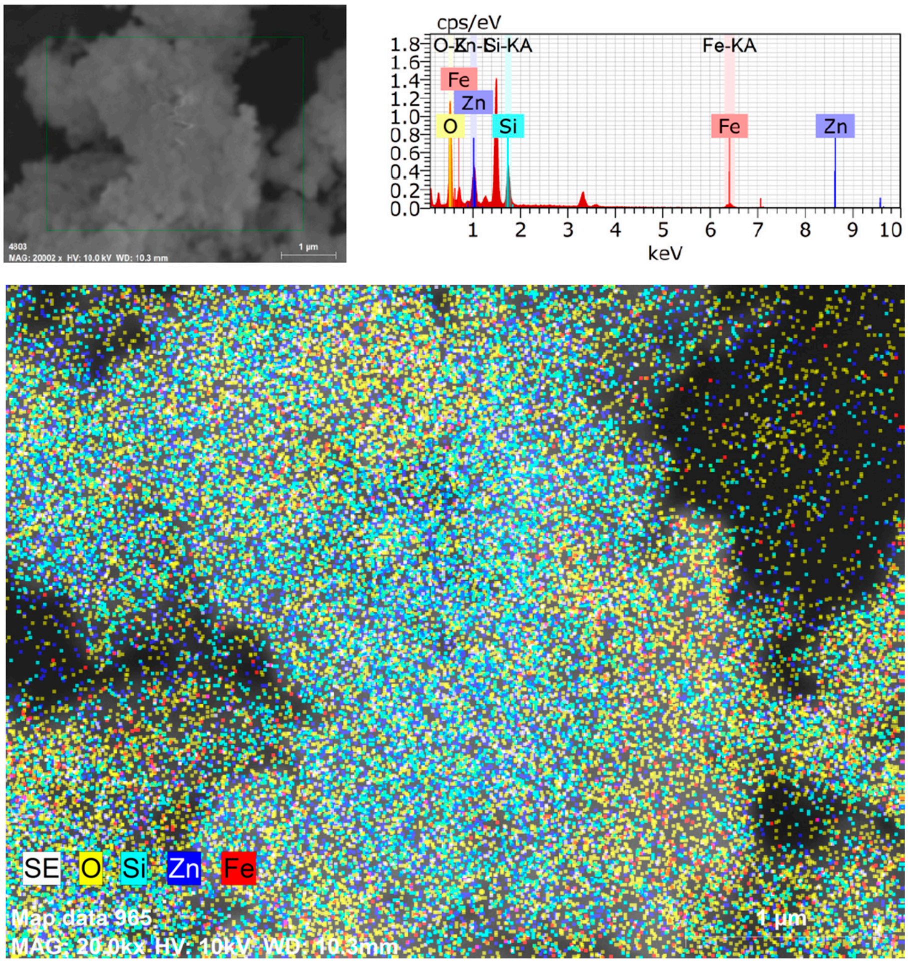

Figure S3. Energy-dispersive X-ray spectroscopy of Fe_3O_4@SiO_2@ZnO

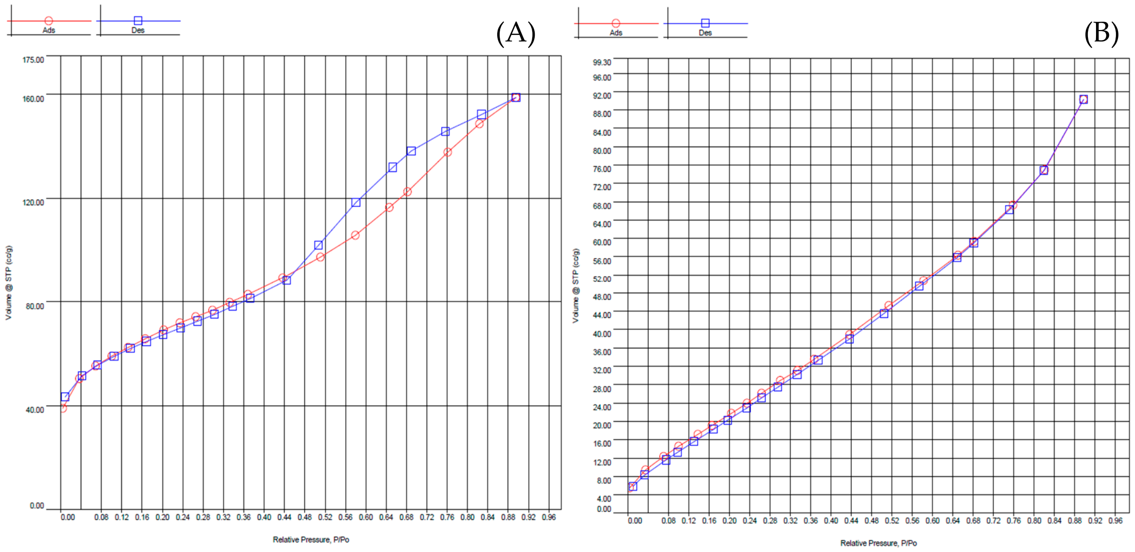

Figure S4. N_2 adsorption-desorption isotherms of synthesized nanocomposites. (A) SPION@SiO_2@TiO_2, (B) SPION@SiO_2@ZnO
